# The formation pattern, causes, and governance of network public opinion on university emergencies

**DOI:** 10.3389/fpubh.2024.1367805

**Published:** 2024-08-23

**Authors:** Xiaoning Gao, Zhuoya Li, Ke Zhang, Chongwu Bi

**Affiliations:** ^1^School of Information Management, Zhengzhou University, Zhengzhou, China; ^2^Research Center of Date Science, Zhengzhou, China

**Keywords:** university emergencies, network public opinion, network public opinion field theory, formation, cause, governance

## Abstract

**Background:**

University emergencies, garnering significant public attention and shaping network opinions, pose a crucial challenge to universities’ management and societal stability. Hence, network public opinion on university emergencies is a vital issue. Nevertheless, the underlying mechanism has not been fully explored and cannot be efficiently controlled. This study aimed to explore the formation pattern of network public opinion on university emergencies, analyze its causes, and provide scientific governance strategies for coping with this issue.

**Methods:**

Based on a sample set of 204 cases from the Zhiwei Data Sharing Platform, this study classifies network public opinion on university emergencies into six types and visually analyzes their characteristics: time distribution, subject, duration, and emotion. By integrating the theory of the network public opinion field, this study develops a network public opinion field model of university emergencies to reveal its formation pattern. Furthermore, it analyzes the causes of network public opinion on university emergencies from the perspective of the public opinion lifecycle and proposes corresponding governance strategies.

**Results:**

The sample consisted of 304 cases of real-life public opinion, and the visualization results show that public opinion on mental health and teacher–student safety constitutes the predominant types, accounting for 83.3%. High-occurrence subjects are public universities (88.24%) and students (48%). The most frequent months are July and December. 90.20% of the public opinions have a lifespan of less than 19 days, with an impact index ranging from 40 to 80. The public’s emotional response to different types of public opinion varies, with negative emotions dominating.

**Conclusion:**

This study provides novel insights for understanding their formation and dissemination. It also provides practical implications for relevant departments to govern network public opinion on university emergencies.

## 1 Introduction

Amid higher education reform, new media proliferation, and university management shifts, university emergencies such as examinations, enrollment, and employment conflicts have been on the rise (1, 2). As an important part of public education, university emergencies are prone to attracting significant public attention (3), particularly driven by the online media, leading to an enhanced tendency for public participation therein (4, 5). With the intensification of public opinion, network public opinion on university emergencies is increasingly characterized by diversified content, complex evolution, extreme emotions, and the proliferation of risks (6). The multifaceted risk characteristics of public opinion pose substantial threats to university management, ordered network environment, and education credibility (7). Thus, it is important and urgent to explore the formation and governance.

At present, the formation of network public opinion including the formation principle and process is mainly studied by social network analysis (8). This type of research traverses various academic domains, including information communication and social psychology, exploring significant topics such as the dynamics of opinion polarization (9), the impact of opinion leaders (10), and the evolution of public opinion (11). One of the centers of research related to the evolution of public opinion is the distinction between public and private opinions (12). Individuals may exhibit significant discrepancies between their private and expressed opinions due to a multitude of factors, including social normative pressures (13), misbehaving individuals (14), and sociocultural influences (15). Failing to acknowledge these discrepancies is likely to have significant ramifications, such as the Arab Spring movement, and the fall of the Soviet Union.

Network public opinion on university emergencies is a weathervane for all segments of the university system (6). It mainly refers to the collective attitude, opinions, and emotions held by Internet users toward hot topics triggered by university emergencies within a certain period. Consequently, this study must encompass both private and public opinions in a comprehensive manner.

As a sub-study of network public opinion on emergencies, research related to network public opinion on university emergencies can be traced back to the 1990s. The initial studies were few in number and limited to specific colleges and universities. With the widespread adoption of Internet technology and the expanding university student population, network public opinion on university emergencies has garnered increasing attention. Subsequently, research into the dissemination patterns, influencing factors, monitoring, and early warning mechanisms has made incremental progress. In recent years, social changes and shifts in information communication have heightened the complexity of public opinion formation and escalated public opinion risks. Therefore, existing researchers have initiated a re-examination of the formation and evolution of network public opinion on university emergencies, with a view to identifying effective governance strategies.

Drawing from the extant literature, the current research on network public opinion on university emergencies focuses on four categories. The first category has focused on the evolution mechanism of network public opinion on university emergencies. Related studies employ various modeling techniques to facilitate intelligent simulation (16, 17). For example, Qu simulated the evolution of network public opinion on university emergencies using an enhanced SNIDR model to illuminate the dynamic interaction mechanism (16). The second category has explored the factors influencing network public opinion on university emergencies. Different from other types of public opinions to prioritize objective factors (18, 19), network public opinion on university emergencies emphasizes greatly on subjective factors, such as social motivation and information source preference (20, 21). The third category has addressed monitoring network public opinion on university emergencies. In recent years, universities have faced several worrisome trends related to student safety and wellbeing, including violent behavior, cyberbullying, and adolescent suicidality (22). To improve such situations, student social media monitoring programs and university dynamic monitoring systems are widely used to effectively identify and prevent potential problems (23–25). In addition, the fourth category is concerned with the governance of network public opinion on university emergencies (6, 20, 26).

Despite the fact that scholars have approached the issue from various perspectives, there are still gaps in the relevant studies. First, the research perspective covers a wide range of emergencies, while rarely paying attention to university emergencies. Universities play a crucial role in public health as a subsystem of the social system and the frontline of ideological dissemination. Existing research has mainly focused on network public opinion on emergencies, but it has failed to effectively incorporate the unique background of university emergencies. Consequently, the research insights cannot fully address network public opinion on university emergencies.

Second, research on the causes of network public opinion on university emergencies has not been sufficiently comprehensive. The formation and dissemination of public opinion is not a simple linear information transmission model, and the complexity of its evolutionary causes is increasing with the evolution of the new media landscape. Despite abundant research on public opinion formation, the existing literature often narrowly focuses on one aspect of its spread, neglecting a comprehensive view of its entire lifecycle (27). Furthermore, it is necessary to explore the interaction among the components of public opinion diffusion.

Third, an entry point and basic theories for exploring network public opinion on university emergencies are lacking. Most studies have considered network public opinion on university emergencies as a whole and have rarely explored its classification. In addition, many scholars have proposed governance strategies from the subject’s perspective, ignoring the internal development characteristics of public opinion. These circumstances have led to a lack of focus on relevant public opinion governance strategies and further hindered in-depth exploration of the field.

To fill these gaps, this study aimed to construct and analyze the network public opinion model by combining data visualization and the theory of network public opinion field and to explore the causes and governance strategies of network public opinion on university emergencies. First of all, this study visually analyzed the network public opinion on university emergencies from the dimensions of type, time, subject, and emotion based on 204 real-life cases. Second, based on the results of the analysis, this study integrated the theory of the network public opinion field to develop a network public opinion field model of university emergencies. By exploring the dominant fields and interactions at different periods of the public opinion lifecycle, the causes of network public opinion on university emergencies were analyzed. Third, from the entire lifecycle perspective, this study proposes periodization governance strategies to enhance the efficiency of network public opinion governance.

The remainder of this paper is organized as follows: Section 2 describes the sample data collection process, including data source, data selection, and extraction. Section 3 reveals the analysis results based on the sample data set from various angles. Section 4 proposes a network public opinion field model of university emergencies to present its formation mechanism and explores its causes and governance strategies. Section 5 concludes the theoretical and practical implications as well as the limitations.

## 2 Materials and methods

### 2.1 Data source and selection

#### 2.1.1 Data source

This study employs microblogging data provided by the “Zhiwei Data Sharing Platform.”^1^

Social media have emerged as a pivotal medium for the dissemination of public opinion, facilitating access to diverse essential information, including opinions and emotions (28). As the number of users and the influence of the platform have increased, Weibo has become the primary center for the dissemination of online public opinion in China (29). However, frequent upgrades have made it increasingly challenging to crawl their data, prompting some scholars to utilize third-party social media opinion aggregation platforms, such as the Zhiwei Data Sharing Platform (30). This platform provides an evaluation system for the influence of network public opinion based on big data technology, integrating social media data to effectively present current and trending topics. Furthermore, the platform employs rigorous and uniform criteria for incorporating public opinion events: (1) Achieving a high volume of dissemination within a brief period of time. (2) Maintaining a consistent volume of dissemination over an extended period. (3) Stimulating heated debates on online social media. These criteria effectively enhance the credibility and accuracy of the data. Therefore, it was deemed reasonable to use the Zhiwei Data Sharing Platform as the data source for this study.

#### 2.1.2 Data selection

This study employed a keyword maximization search method to gather data. Considering the main subjects of public opinion on university emergencies, the following keywords were used for searching: “college,” “campus,” “university,” “student,” “postgraduate,” “teacher,” “professor,” and “faculty.” Furthermore, the search timeframe was set from 1 January 2015 to 31 December 2022.

The following inclusion criteria were formulated to ensure the validity of the sample data:

(1) The subject of the incident should be the university group or the university itself. Even if the site of the emergency is off campus, the entities involved are university-affiliated groups or the universities themselves.
(2) The incident occurred during the period of higher education. This criterion ensures that the research object is a university emergency.
(3) The incident was sudden. Suddenness, which is one of the main characteristics of university emergencies, is also a crucial aspect of network public opinion and a challenge in its governance (31).
(4) The negative impact of the incident was an imbalance in order (32). The incident had a certain impact on the university or community, disrupting the campus work or public opinion environment.

**Figure 1:**
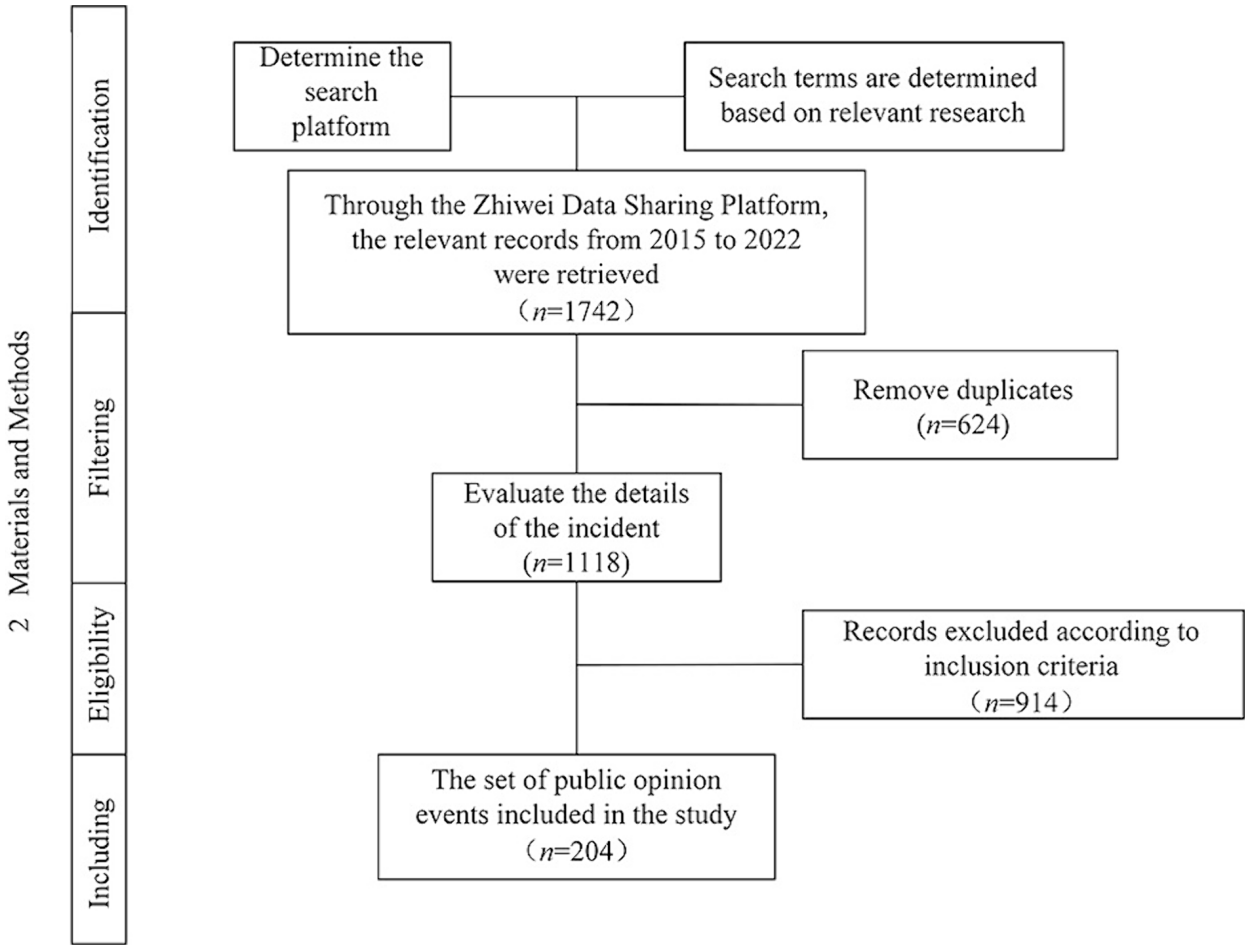
Sample data selection process.

Following the above selection criteria and sample data selection process in Figure 1, 204 valid samples were obtained from the Zhiwei Data Sharing Platform.

### 2.2 Data extraction

Based on the obtained sample data, the following fields were extracted for subsequent analysis:

(1) Occurrence time of public opinion. The year of occurrence and the month of public opinion in the sample set were extracted. These data reveal the distribution of public opinion incidents in the time dimension.
(2) Type of university involved. The universities involved in the sample set were divided into public universities, private universities, and others according to their attributes. These data reveal the distribution of universities involved in public opinion incidents.
(3) Impact index of public opinion. The impact index within the Zhiwei Data Sharing Platform is calculated by summing the dissemination effects across self-media (Meibo and WeChat) and online media and then normalizing the resulting sum. The value of these data ranges from 0 to 100, indicating the dissemination intensity and spread of public opinion incidents on Internet platforms.
(4) Subjects of public opinion. The subjects involved in public opinion were divided into student, teacher, school, student–teacher, student–school, and others based on their relationships. These data can be used to analyze the subjects involved in public opinion incidents.
(5) Titles and abstracts of public opinion. The titles and news abstracts of each public opinion on the platform were preprocessed to obtain effective vocabulary. These data offer a rapid comprehension of the progression of public opinion incidents and the focal points of public interest.
(6) Public opinion commentary. Public opinion leaders are pivotal nodes in the dissemination of public opinion (10). These users are less susceptible to external influence when expressing their opinions, and their comments are primarily reflective of their private opinions. “Like” is a significant indicator for gauging social consensus (33). High “like” comments can be regarded as representatives of public opinion due to their extensive dissemination and acceptance. Therefore, this study comprehensively collected opinions from opinion leaders or highly popular comments on Weibo platforms, in order to ensure the comprehensiveness and accuracy of data analysis. These data can reveal the public’s views and attitudes toward public opinion incidents.
(7) Duration of public opinion. With days as the unit of time, the duration of public opinion on the platform was extracted. These data reveal the durations of active public opinion incidents on Weibo.

## 3 Results

### 3.1 Classification and characteristics of network public opinion on universities emergencies

#### 3.1.1 Classification of network public opinion on universities emergencies

Classifying the types of network public opinion on university emergencies is a prerequisite for studying their evolution and governance. This is conducive to clarifying evolutionary patterns and providing rapid and targeted responses and governance.

**Figure 2:**
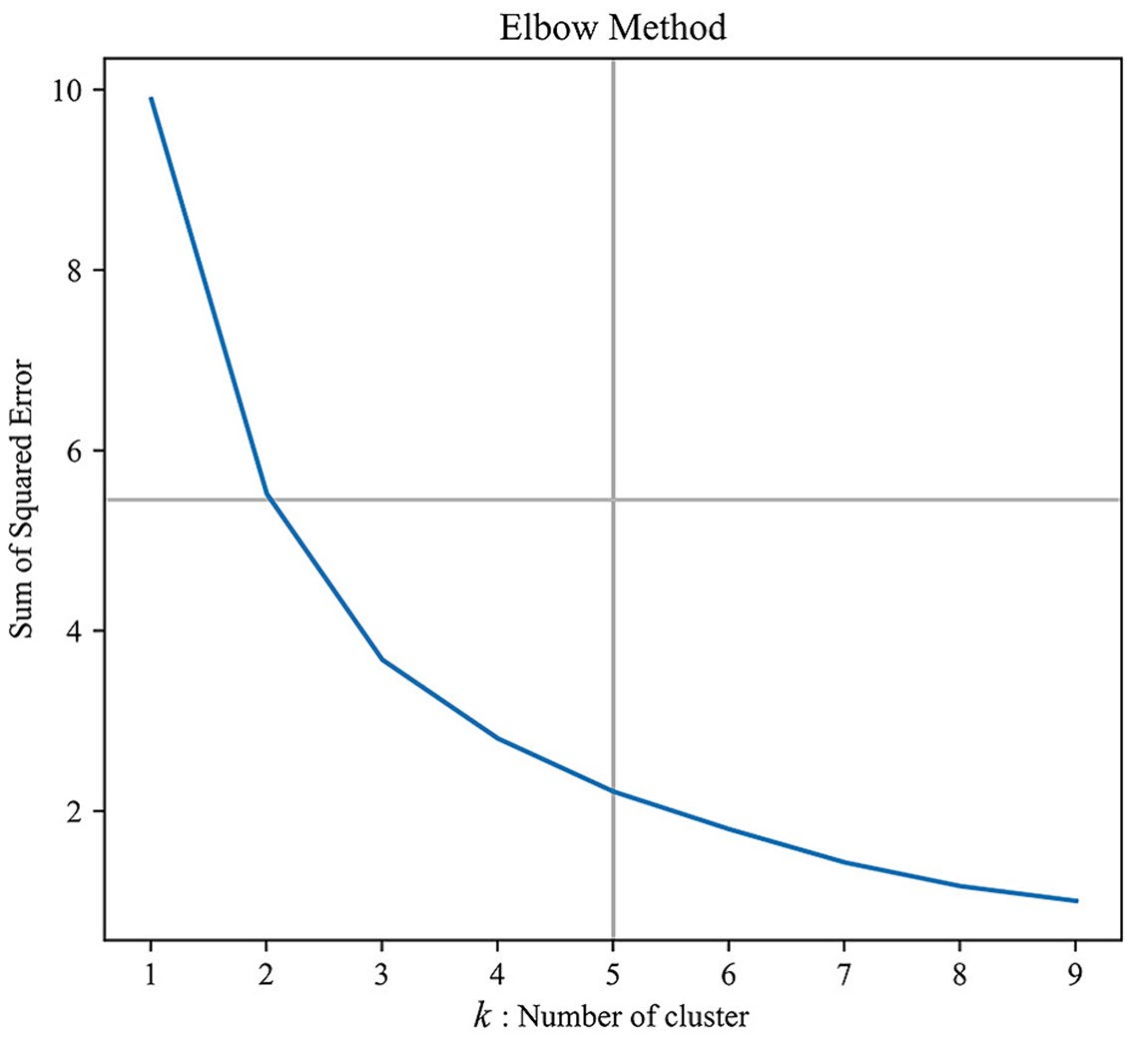
Optimal number of clusters.

A semantic analysis of the themes in the sample data revealed that the optimal number of clusters—and thus the optimal classification of the types of network public opinion regarding university emergencies—was four (Figure 2).

In addition, the sample data included a small number of public opinions on natural disasters and social and political issues. Hence, this study developed six categories of network public opinion on university emergencies, as shown in Table 1.

(1) Network public opinion on natural disaster emergencies. It refers to the university network public opinion triggered by natural disasters such as earthquakes, floods, typhoons, and blizzards. It is characterized by suddenness and unpredictability.
(2) Network public opinion on sociopolitical emergencies. It refers to the university network public opinion triggered by university groups in the fields of international situations, religious beliefs, and national sentiments. The incidence of such network public opinion on university emergencies is low but politically charged. It is characterized by high cohesion among opinion subjects, extensive diffusion of public opinion, and substantial social influence.
(3) Network public opinion on public service emergencies. It refers to a university network public opinion triggered by improper campus management, including public health, network information security, management systems, and decision-making. The incidence of such network public opinion on university emergencies is high; however, the nature and impact indices vary widely. It is characterized by diversity and high incidence.
(4) Network public opinion on teacher–student safety emergencies. It refers to the university network public opinion triggered by campus accidents and students’ and teachers’ personal safety. As university teachers and students are mostly young people with low awareness and ability to cope with danger, these public opinions have typical age characteristics.
(5) Network public opinion on academic security emergencies. It refers to the university network public opinion triggered by teaching accidents and academic fraud by students and teachers. This particular type not only endangers the university’s development in the academic world but also leads to a credibility crisis for the entire education system.
(6) Network public opinion on mental health emergencies. It refers to the university network public opinion triggered by students’ inappropriate behavior and teachers’ moral transgressions. This particular type is mainly caused by human factors. The subject’s behavior, which is somewhat hidden, is induced by some opportunities.

**Table 1:** Types of network public opinion on university emergencies.

Category	Type	Segmentation
A	Natural disasters	A Earthquakes, floods, etc.
B	Sociopolitics	B Ideology, ethnic issues, etc.
C	Public services	C1 Public health: canteen hygiene.
		C2 Network information security: campus network failures and network attacks.
		C3 Management system: charges, administrative disposal.
D	Teacher–student safety	D1 Laboratory accidents, dormitory fires, campus public facilities.
		D2 Personal health: sudden death, loss of contact.
E	Academic safety	E1 Teaching accidents: examination accidents, improper study style.
		E2 Academic fraud.
F	Mental health	F1 Students’ inappropriate words and behaviors.
		F2 Teachers’ moral corruption.

The above six types cover all the current network public opinion on university emergencies. In reality, however, different types of network public opinion on university emergencies are not clearly defined and completely independent; under certain scenarios, they may overlap or even transform into each other. Therefore, it is necessary to comprehensively govern public opinion in line with its actual development.

#### 3.1.2 Characteristics of network public opinion on university emergencies

**Figure 3:**
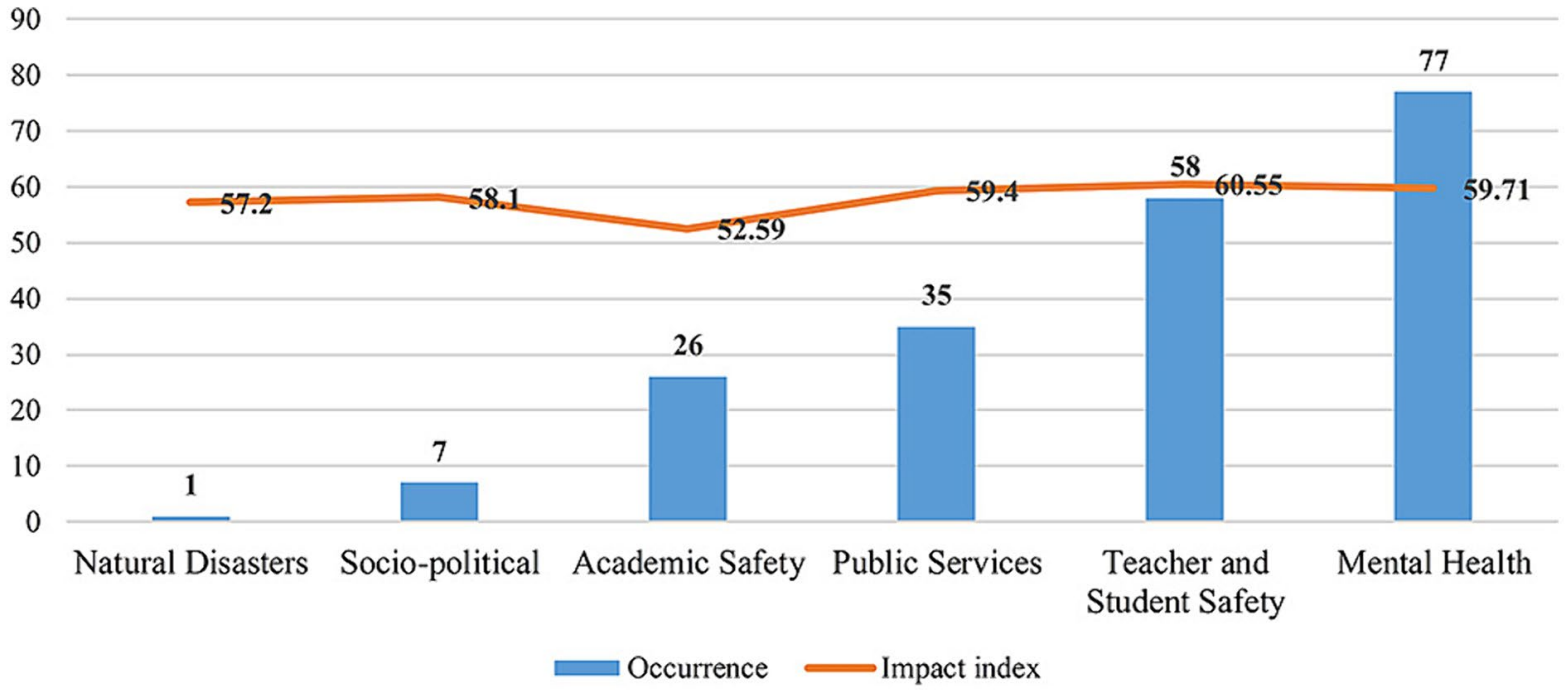
Occurrence volume and impact index of network public opinion on university emergencies.

Understanding the characteristics of different types of network public opinion on university emergencies can help establish or improve public opinion response mechanisms. Hence, this study analyzed the occurrence volume and impact index of different types of public opinion incidents to identify their characteristics, as shown in Figure 3.

In terms of the number of occurrences, mental health network public opinion and teacher–student safety public opinion have the highest occurrence, with both types of public opinion accounting for 83.30% of the total sample size, followed by public service, academic ethics, sociopolitics, and natural disasters. In terms of the impact index, network public opinion on teacher–student safety was the highest, followed by mental health, public service, sociopolitics, natural disasters, and academic safety. Hence, mental health and teacher–student safety were the two commonly occurring types of network public opinion with a high impact index, and they should be considered priorities in the governance of network public opinion on university emergencies.

### 3.2 Time distribution of network public opinion on university emergencies

Network public opinion on university emergencies is characterized by substantial dynamic evolution. Exploring its evolution process helps to understand the relevant elements affecting public opinion (34) and provides a reference for public opinion governance. Hence, this study analyzes the time distribution of network public opinion on university emergencies.

**Figure 4:**
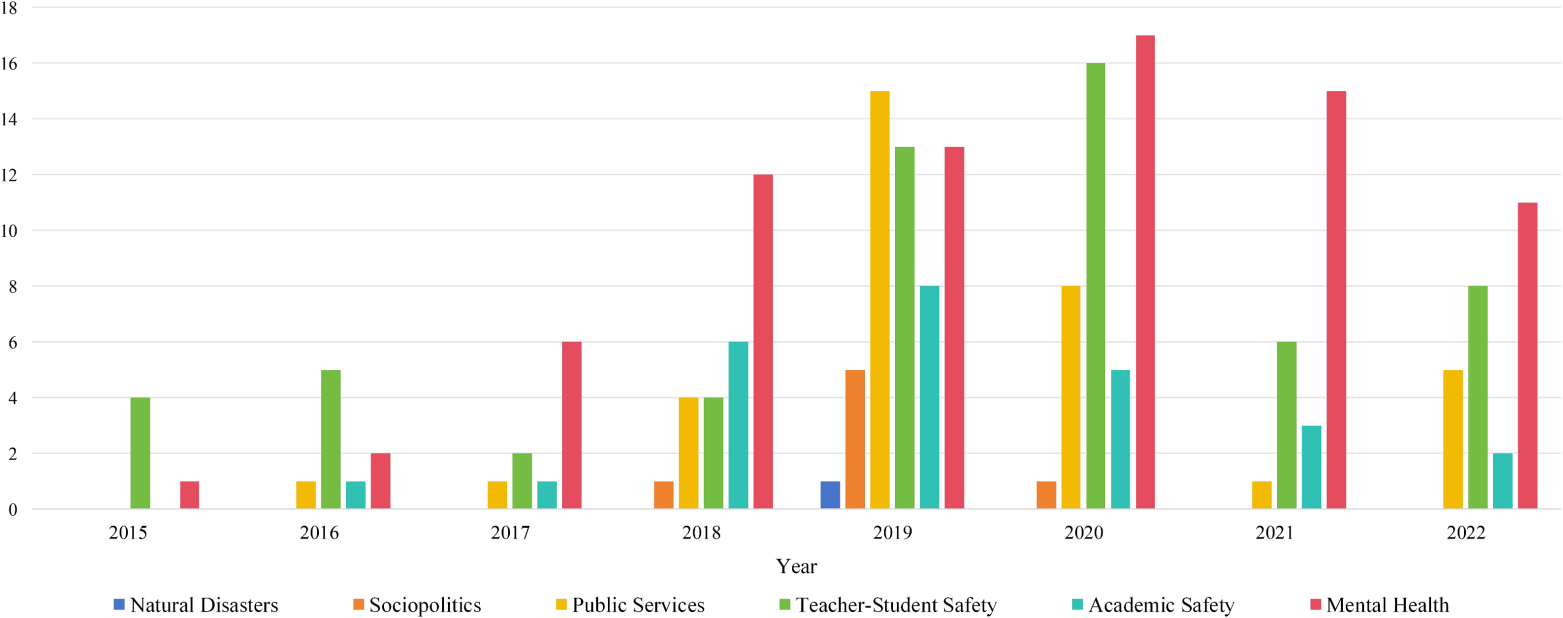
Annual distribution of network public opinion on university emergencies.

First, this study examined the annual occurrence changes of different types of network public opinion on university emergencies, as shown in Figure 4.

The total number of network public opinion on university emergencies showed an overall “inverted U-shape.” The growth was slow from 2015 to 2018. It peaked between 2019 and 2020 and showed a downward trend from 2021 to 2022. Moreover, the overall fluctuation of network public opinion on natural disasters and sociopolitics was relatively small, indicating that the attention to such incidents was stable. Network public opinion on public services, teacher–student safety, and academic safety varied sharply by year. In recent years, public opinion on mental health has increased, quickly becoming the most common type of network. This indicates that network public opinion on mental health is becoming increasingly prominent.

The growth from 2019 to 2020 is closely related to the COVID-19 pandemic. Universities are considered as one of the “main battlefields” of epidemic prevention and control (35). During the COVID-19 pandemic, universities implemented a series of stringent lockdown measures, necessitating an abrupt adaptation of existing education systems to a new online teaching environment (36). Due to the disruption of teaching order, internal conflicts within universities intensified, leading to a comprehensive increase in occurrences from 2019 to 2020. As the pandemic waned and university management was optimized, the occurrences declined. Beyond physiological harm, the COVID-19 pandemic has also triggered widespread psychological crises (37, 38). The university population, particularly freshmen, exhibits heightened vulnerability in terms of mental health, with notable elevations in stress levels, anxiety, and depressive thoughts during the pandemic (39, 40). As stress levels escalate, individuals inevitably project their burdens onto social media (41), creating a vicious cycle where the overwhelming influx of information exacerbates mental health problems (42, 43). Consequently, public opinion on mental health still remains high after the COVID-19 pandemic (44), and it has gradually become the mainstream public opinion on university emergencies.

**Figure 5:**
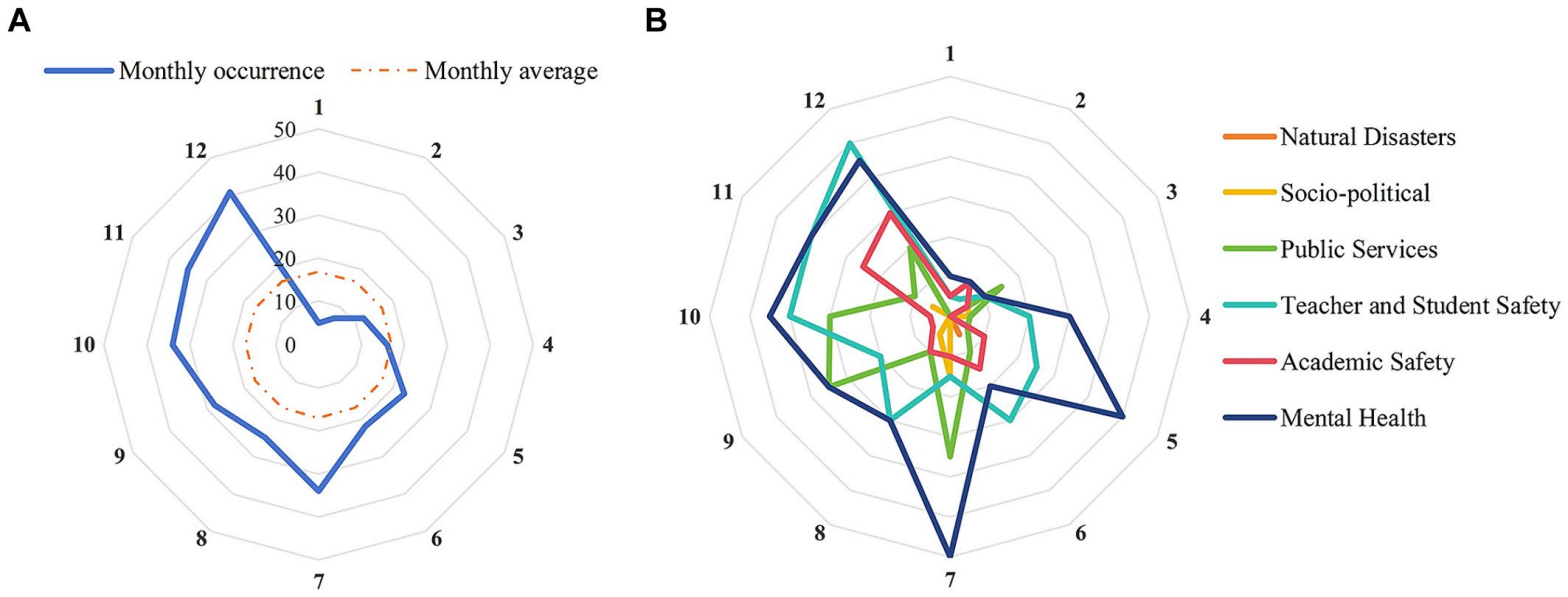
Monthly distribution of network public opinion on university emergencies. **(A)** Overall distribution. **(B)** Distribution of each type.

Second, this study analyzed the monthly occurrence changes of different types of network public opinion on university emergencies, as shown in Figure 5.

The monthly average occurrence of 17 cases as the basis shows that the occurrence of network public opinion on university emergencies varied greatly by month (Figure 5A). It mainly occurred in the second half of the year and had a double-peak distribution in July and December. Furthermore, the emergence of network public opinion on university emergencies is intricately linked to the university’s management cycle (Figure 5B). Public opinion is usually accompanied by various university activities with high mobility and pressure, including festivals and holidays, awards and assessments, major examinations, and campus recruitment. These activities are prone to attracting public attention and discussion, thereby generating relevant network public opinion.

### 3.3 Subject of network public opinion on university emergencies

**Figure 6:**
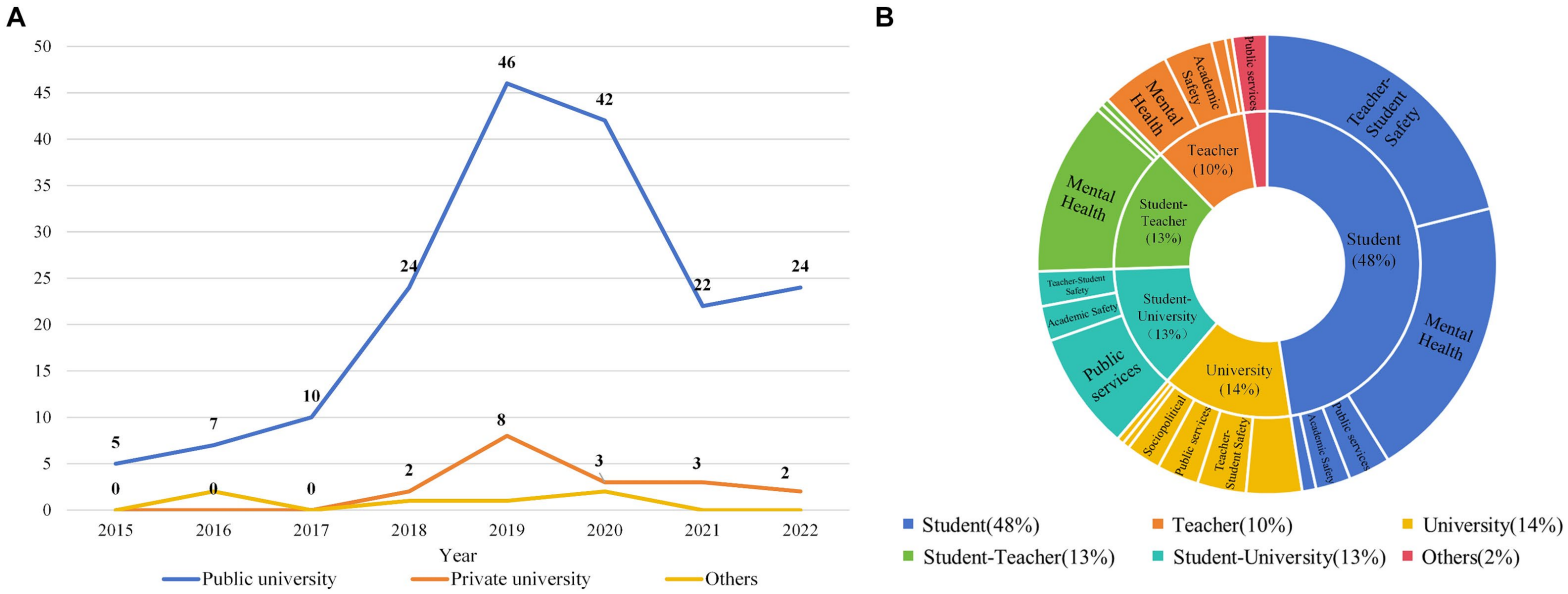
Subject distribution of network public opinion on university emergencies. **(A)** Proportion of different types of universities. **(B)** Proportion of different public opinion subjects.

Network public opinion dissemination is the outcome of a subject’s behavioral choices in a specific cyberspace (45). Students, teachers, and universities participate in university education, and they are also the subjects of network public opinion on university emergencies. Hence, this study analyzed subject characteristics to understand the behavior and role of different subjects in the development of network public opinion on university emergencies, as shown in Figure 6.

The occurrence rate of network public opinion on university emergencies in public universities stands at 88.24%, significantly exceeding that in private universities (Figure 6A). The reasons for this finding are two. First, China’s higher education system is dominated by public universities and supplemented by private universities. The number of public universities significantly exceeds that of private universities. Second, public universities place more emphasis on cultivating student information quality, leading to higher levels of online participation and dominance among students at public universities. This results in a high degree of social concern for public universities, which makes it easier for public opinion to form.

The occurrence of network public opinion on university emergencies was the highest among students, accounting for 48% of the total, followed by school, student–teacher, and student–school (Figure 6B). In addition, different subjects are often associated with specific types of public opinion, which is related to the subject activities and group characteristics. Students, being inclined toward having an active mind and being open to novelty, often lack social practical experience and are prone to the influence of harmful information (46). Consequently, they frequently become ensnared in public opinion, regarding issues such as teacher–student safety and mental health. University teachers, often facing substantial professional pressure (47), have experienced a gradual decline in their mental wellbeing in recent years (48, 49). Consequently, public opinion involving teachers has primarily focused on mental health. Universities are social and cultural centers with talent training, scientific research, and innovation as their main activities. Therefore, public opinion related to public services and academic safety at universities is more likely to evoke widespread social discussion.

### 3.4 Duration of network public opinion on university emergencies

**Figure 7:**
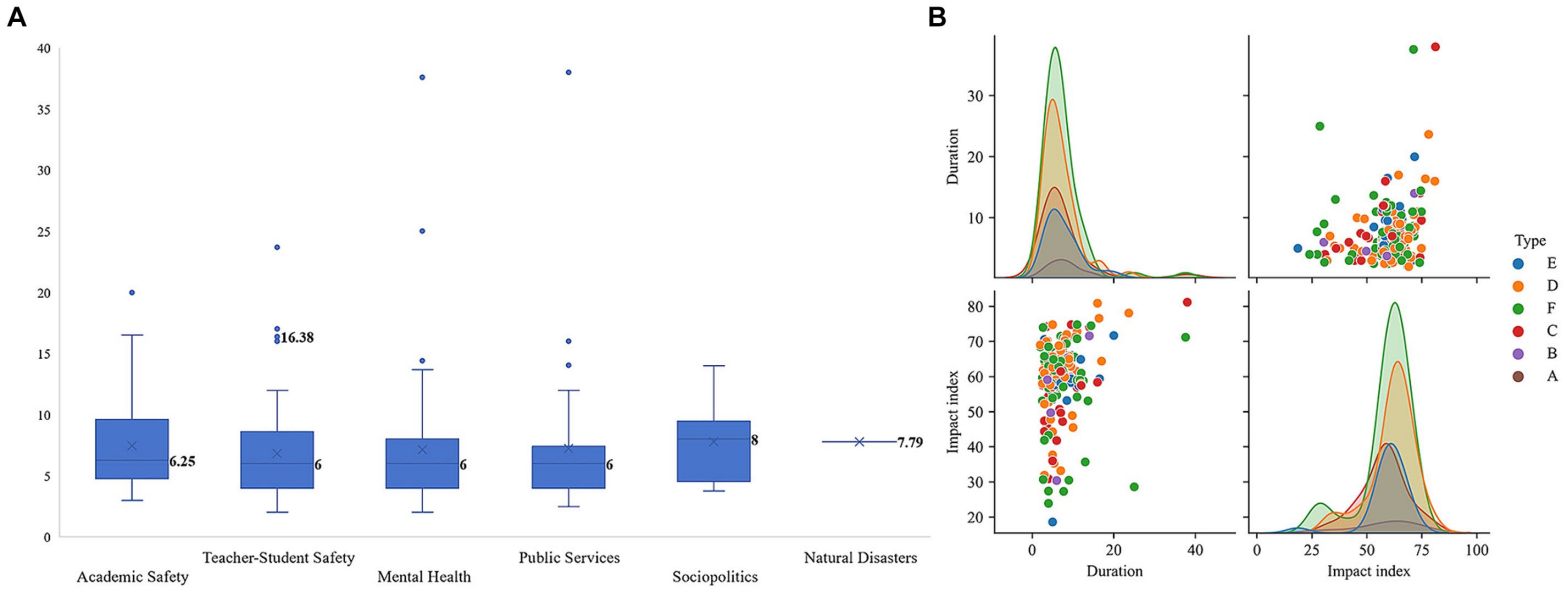
Analysis of the duration of network public opinion on emergencies in colleges and universities. **(A)** Distribution of duration. **(B)** Relationship between duration, type, and impact index.

Duration is an important variable in the study of public opinion (50). Hence, this study analyzed the duration distribution to understand social attention toward different types of public opinion, as shown in Figure 7.

The duration of the six types of network public opinion on university emergencies was centrally distributed over 4–9 days, with medians of 6.25, 6, 6, 6, 8, and 7.79 (Figure 7A). This indicates that most network public opinion on university emergencies tends to last for approximately 1 week.

The distribution of network public opinion on university emergencies in terms of duration and impact index conformed to a normal distribution (Figure 7B). It was found that 90.20% of public opinion samples remained stable at 0–19 days, and their impact index remained within the range of 40–80. Furthermore, the correlation coefficient between duration and impact index was 0.211, with a significance value of 0.002 < 0.001 (Table 2). Therefore, a significant weak positive correlation was observed between the duration and impact index of network public opinion on university emergencies.

**Table 2:** Correlation analysis of duration with impact index.

		Impact index	Duration
Impact Index	Pearson’s correlation	1	0.211
	Sig. (2-tailed)		0.002
	N	204	204
Duration	Pearson’s correlation	0.211	1
	Sig. (2-tailed)	0.002	
	*N*	204	204

**Figure 8:**
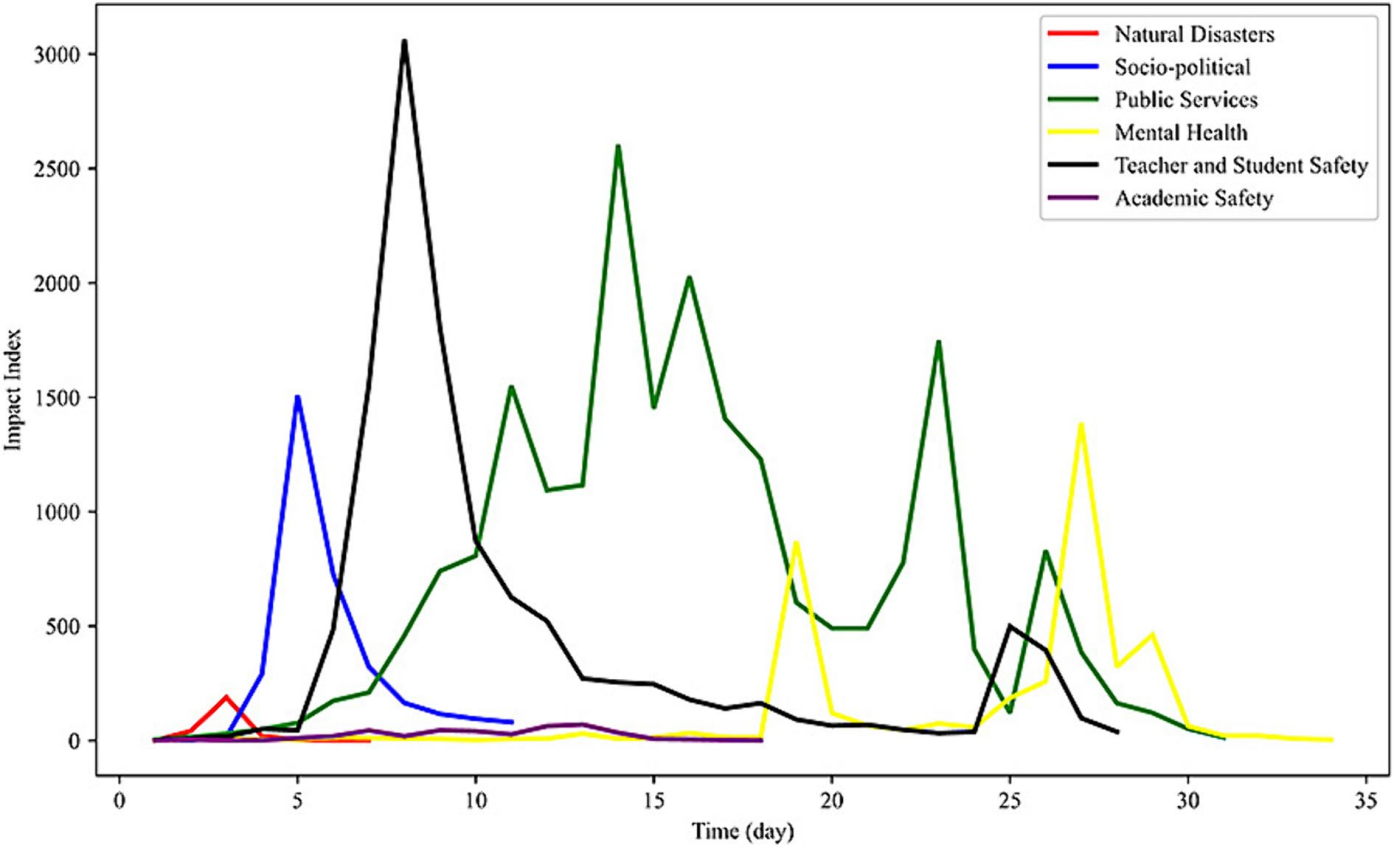
Evolution trend of network public opinion on university emergencies.

This study further explored the factors that affect the duration of public opinion. Duration is a quantitative indicator and measure of the public opinion lifecycle that includes multiple periods of public opinion development. It can be divided into three periods (51), four periods (52), five periods (53), and six periods (54) based on different segmentation methods. Employing a public opinion lifecycle framework can facilitate the analysis of changes in the duration of public opinion. This study selected the longest-lasting cases under different types and investigated the corresponding trend in public opinion evolution, as shown in Figure 8.

In summary, this study divided the network public opinion on university emergencies into five periods: brewing, diffusion, outbreak, dissipation, and calming. During the diffusion period, the curves of public opinion on natural disasters, social politics, and academic safety steadily declined, whereas those pertaining to public services, mental health, and teacher–student safety resurged. The first three types of public opinion had a relatively short duration, whereas the last three types had a longer duration. Therefore, it can be inferred that the dissipation period is crucial in influencing the duration of network public opinion on university emergencies.

### 3.5 Emotion analysis of network public opinion on university emergencies

**Figure 9:**
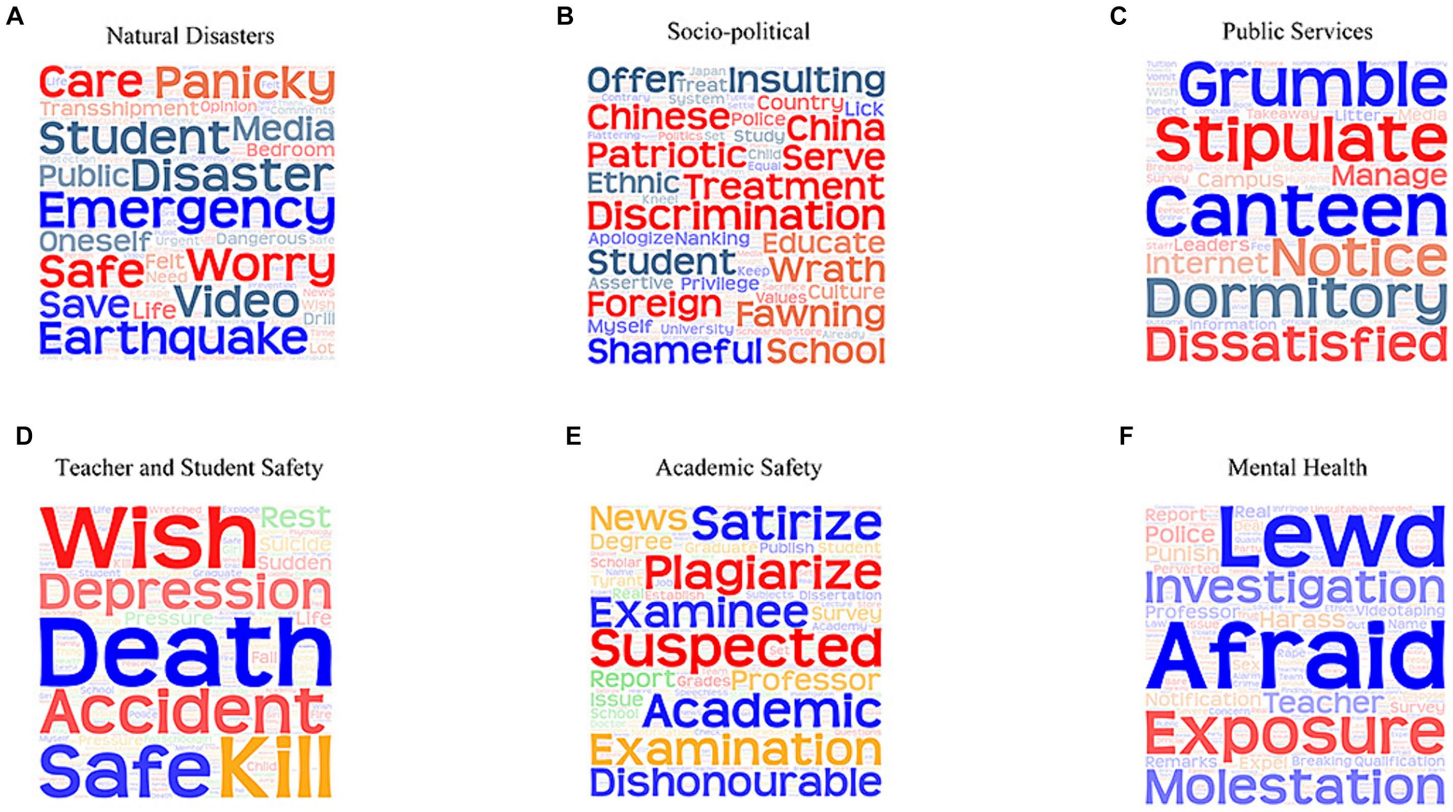
Word cloud of network public opinion on university emergencies. **(A)** Natural disasters. **(B)** Socio-political. **(C)** Public service. **(D)** Teacher and student safety. **(E)** Academic safety. **(F)** Mental health.

The dissemination of public opinion is also a process of transforming public emotions (55). Understanding the emotions of network public opinion on university emergencies aids in exploring the evolution mechanism (56). Hence, this study extracted keywords expressing obvious emotional tendencies in microblog comments to construct emotional word clouds, as shown in Figure 9.

Emotions toward different types of network public opinion on university emergencies varied, basically dominated by negative emotions. The keywords in the word cloud of public opinion on natural disasters (Figure 9A) include “earthquake,” “student,” “safe,” “panicky,” and “worry,” reflecting the public’s concern for student safety and fear of disasters. The keywords in the word cloud of sociopolitical public opinion (Figure 9B) include “foreign,” “discrimination,” “patriotic,” “China,” “fawning,” “insulting,” and “shameful,” reflecting the public’s patriotic feelings and anger toward the trend of worshiping foreign countries. The keywords in the word cloud of public service opinion (Figure 9C) include “canteen,” “dormitory,” “notice,” “dissatisfied,” “stipulated,” and “grumble,” reflecting the public’s dissatisfaction with campus management. The keywords in the word cloud of public opinion on teacher–student safety (Figure 9D) include “safe,” “wish,” “kill,” “depression,” “pressure,” and “death,” reflecting the public’s grief over the accident casualties and prayers for the deceased. The keywords in the word cloud of academic safety opinion (Figure 9E) include “suspect,” “satirize,” “plagiarize,” “academic,” and “dishonorable,” reflecting the irony and helplessness of the public toward academic misconduct. The keywords in the word cloud of public opinion on mental health (Figure 9F) include “afraid,” “molestation,” “lewd,” “harass,” and “notification,” reflecting the public’s fear of sexual harassment and molestation.

The reason for this is that the public’s expression of emotions in network public opinion depends on their own risk perception (57). Individuals generate corresponding emotional feedback based on the risk characteristics brought by different types of network public opinion. Among them, risk perception is most likely to elicit negative emotions (58).

**Figure 10:**
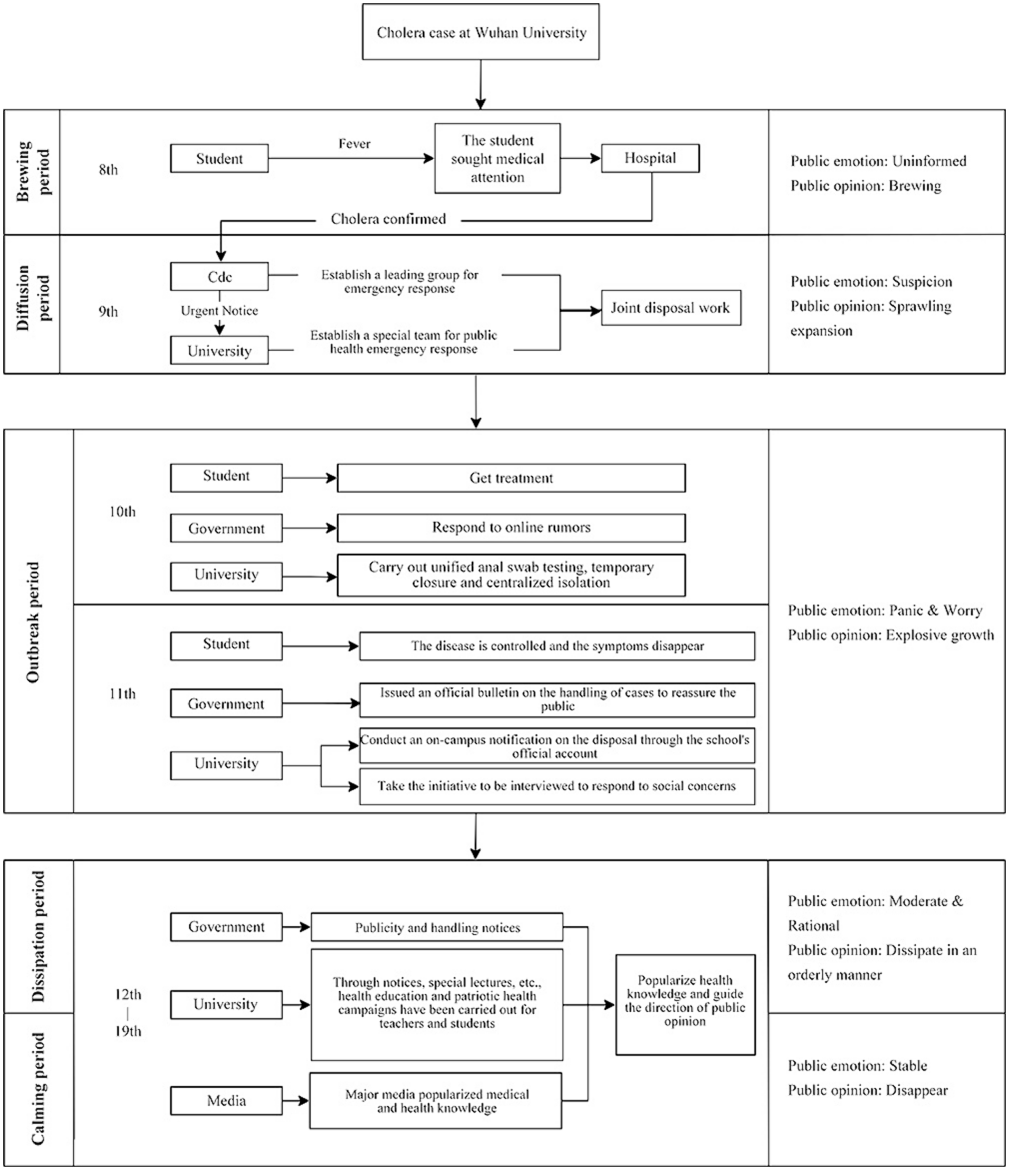
Case of cholera at Wuhan University.

This study further explored the relationship between emotions and the public opinion lifecycle. The network public opinion of “cholera cases in Wuhan University” was taken as the research case. In this case, the impact index was as high as 74.8. However, the duration was only 9.5 days. Therefore, the correlation between impact index and duration may not have interfered with the analysis to a certain extent. Hence, this study reviewed the entire case to analyze the changes in emotions and the evolution trend of public opinion, as shown in Figure 10.

Public opinion expressed different emotional tendencies in different periods. On 8 July, most of the public was uninformed, and public opinion was in the brewing period. On 9 July, public opinion diffused amid public suspicion of cholera. From 10 to 11 July, a case of cholera was identified, which led to a rapid outbreak of public opinion. From 12 to 19 July, with various management departments demonstrating efficient joint governance capabilities, the public panic was alleviated, and public opinion dissipated accordingly. The general public’s emotional changes drive the cyclic evolution of network public opinion on university emergencies. This is because the development process of network public opinion on university emergencies involves public information demand and information feedback. Emotional demand is also a part of information demand (59). To satisfy their emotional needs, netizens take the initiative to contact the public and express their emotions; therefore, their emotional demand is crucial in influencing the impact index of public opinion.

## 4 Discussion

The results above indicate that network public opinion on university emergencies has certain regularities in terms of characteristics, time distribution, subject, duration, and emotion. These regularities form a relatively independent network space called the network public opinion field. Based on the theory of the network public opinion field, this study constructs a network public opinion field model of university emergencies. By analyzing the interactions between different fields, this study further examines the underlying causes from a lifecycle perspective. Moreover, related governance strategies are proposed to provide insights into network public opinion on university emergencies.

### 4.1 Network public opinion field model of university emergencies

The popularity of the Internet and new media has shifted public opinion from print media to online, resulting in a network field dominated by social media platforms (60). As a branch of field theory, the network public opinion field refers to a spatial and temporal environment containing several interacting factors that enable public opinion (61). The theory emphasizes how different viewpoints, emotions, and attitudes intersect and collide in a particular public opinion environment. This theoretical framework is highly compatible with the specific social opinion phenomenon of network public opinion on university emergencies.

Network public opinion on university emergencies can be considered the result of the combined influence of multiple factors (62). In the process of formation and dissemination, the influencing factors of network public opinion on university emergencies are interrelated, attracting the voices of students, faculty, administrators, and external stakeholders. These voices intertwine and collide in cyberspace, forming a network public opinion field of university emergencies containing multiple subfields. An evident correspondence exists between the subfields and influencing factors, and the different subfields share an interaction and constraint relationship. The network public opinion field presents the characteristics of plurality, interactivity, and dynamics with the development of network public opinion on university emergencies. By emphasizing these characteristics, the theory of the network public opinion field provides a powerful theoretical tool and analytical framework for understanding, analyzing, and responding to network public opinion on university emergencies.

**Figure 11:**
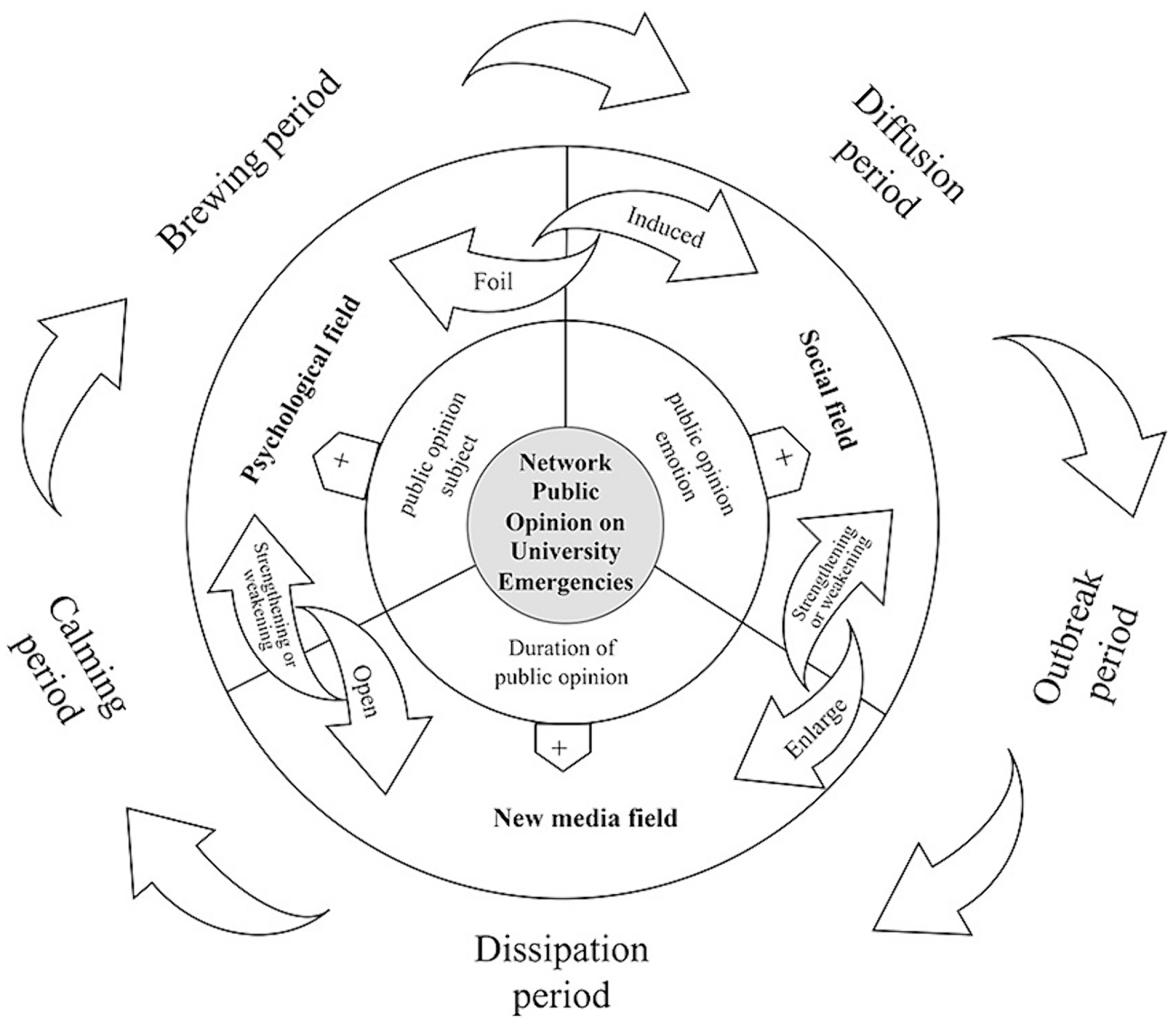
Network public opinion field model of university emergencies.

Drawing from the theory of the network public opinion field, this study identifies key factors shaping network opinion on university emergencies and establishes a corresponding model (Figure 11). The model divides the whole network public opinion field of university emergencies into three subfields: psychological field, social field, and new media field. This reveals the interaction within and between each subfield from the perspective of the public opinion lifecycle.

With respect to each field, the relationship with its elements is as follows:

(1) Psychological field—subjects of public opinion. The psychological field of network public opinion on university emergencies refers to the density of people and frequency of communication in the same space. The higher the crowd density and interaction frequency, the stronger the subject’s dissemination effect in the psychological field. Universities, teachers, and students are key subjects in the evolution of network public opinion on university emergencies. They converge in their cultural communication, living space, and interests and tend to have the same opinion, which makes it easy for them to resonate with each other’s emotions and cooperate. This characteristic enables them to easily accumulate public opinion both online and offline, which spreads further due to other influencing factors. Therefore, the subject of public opinion is the key factor in the psychological field.
(2) Social field—public opinion emotion. The social field of network public opinion on university emergencies refers to the rendering objects and atmosphere of the public opinion environment. The greater the number of rendering objects and the stronger the rendering atmosphere, the stronger the emotion in the social field. The rendering atmosphere consists of various figurative rendering objects such as opinion labels and online slogans. They enhance the communication of a point of view or emotion by stimulating the senses and attracting a high level of public attention. The network public opinion on university emergencies contains rich emotional expressions, and the tendency and intensity of these emotions have a significant impact on the evolution of public opinion (63). Thus, public opinion emotion is a key factor in the social field.
(3) New media field—duration of public opinion. The new media field of network public opinion on university emergencies refers to the openness of the public opinion environment. The higher the openness of public opinion, the longer the duration of public emotion in the new media field. The network public opinion field and society are in a relationship of part and whole. In the early development of network public opinion on university emergencies, small-scale public opinion is connected to the overall social environment through the new media field; as a result, a smooth information channel is established. The openness of the information channel affects the intensity of public information expression in the public opinion environment. High-intensity information expression often awakens other similar issues under public opinion, generating derivative public opinion and lengthening its duration. Therefore, the duration of public opinion is a key factor in the new media field.

Moreover, an interactive relationship exists among all fields. First, network public opinion on university emergencies is noticed and disseminated by subjects of public opinion in the psychological field. Through actions such as liking, commenting, and sharing on social media, public opinion subjects gradually open up the new media field and induce emotion in the social field. Second, under the influence of emotional motivation, the social field creates an atmosphere of emotional attributes, which, in turn, shapes the psychological field and expands the dissemination of the new media field. Third, the new media field interfaces with the psychological and social fields through the information channel. Expanding the information channel means that the new media field is more likely to strengthen the subject’s communication and emotional expression. Conversely, when the information channel narrows, the power generated by the psychological and social fields diminishes. Public attention then shifts to other events, thus shortening the duration of public opinion.

In summary, network public opinion on university emergencies forms and evolves under the interaction within and among subfields. The evolutionary cycle includes five periods: brewing, diffusion, outbreak, dissipation, and calming.

### 4.2 Causes of network public opinion on university emergencies

**Figure 12:**
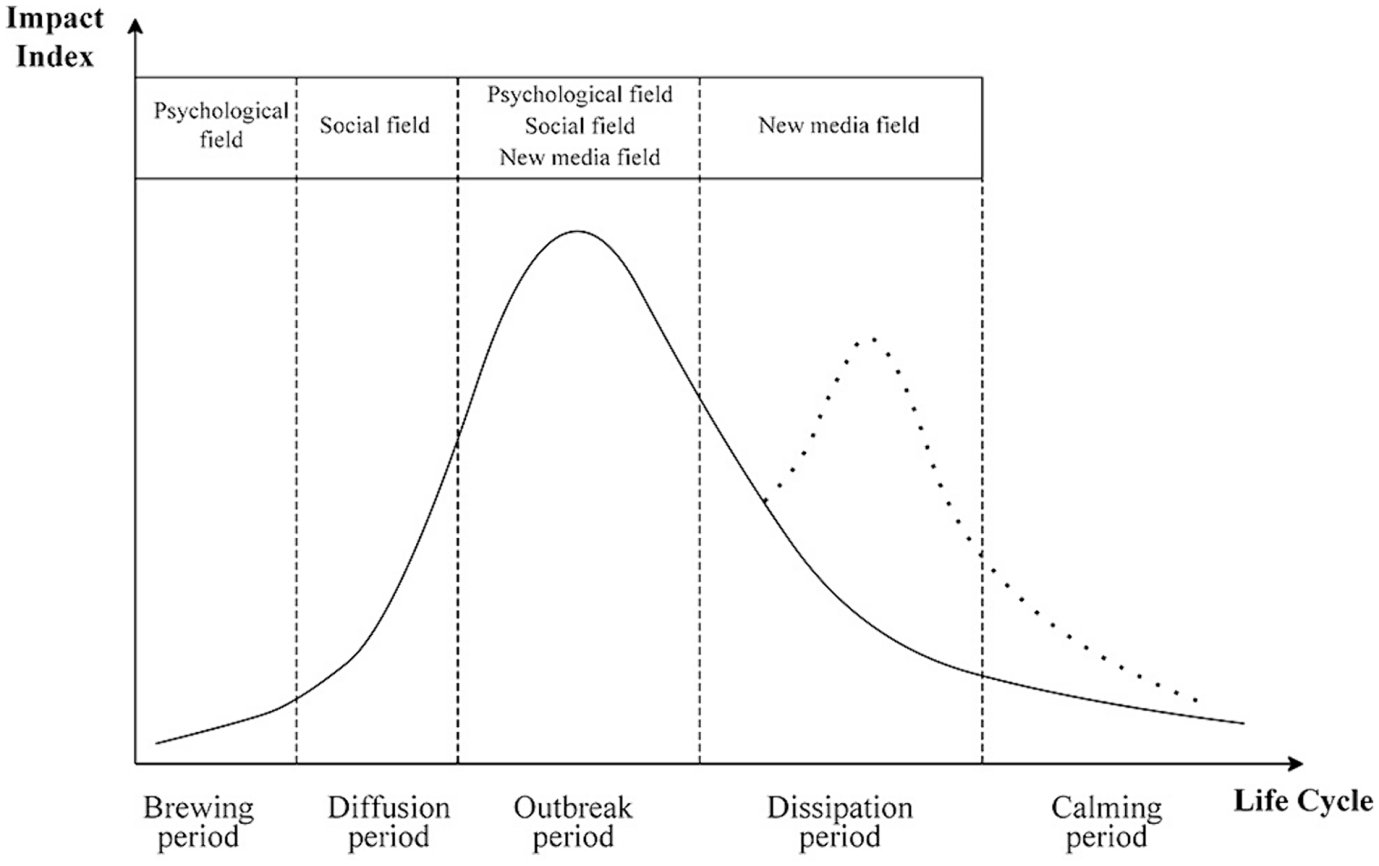
Dominant fields at different periods of network public opinion on university emergencies.

The evolution of network public opinion on university emergencies is closely related to the interactions between the psychological, social, and new media fields. Based on the constructed network public opinion field model of university emergencies, this study clarifies the underlying causes from the perspective of the public opinion lifecycle. The different periods of network public opinion on university emergencies are dominated by specific fields, as shown in Figure 12.

The brewing period is dominated by the psychological field when public opinion has a certain degree of concealment and inducement. The diffusion period is dominated by the social field when the interaction and risk diffusion of public opinion intensify. The outbreak period is dominated by the psychological, social, and new media fields, and the impact index of public opinion reaches its peak at this time. The dissipation period is dominated by the new media field when the direction of the force of the new media field determines the duration of public opinion. The calming period is not dominated by any field, and the whole network public opinion field gradually withdraws from the network environment.

The causes of network public opinion on university emergencies at various periods are explained below.

(1) Brewing period

During the brewing period, network public opinion on university emergencies is mainly concentrated within the university. The analysis results of subjects’ public opinion (Figure 6) show that students are the main group triggering network public opinion on university emergencies. As network public opinion on university emergencies always resonates with university students, it spreads more easily within the university network circles. Therefore, brewing the psychological field is an important basis for transferring network public opinion from university communication to social communication.

(2) Diffusion period

With the increase in impact index, network public opinion on university emergencies enters the diffusion period and is no longer limited to the university; an increasing number of social netizens begin to pay attention to the progress of public opinion, forming a large-scale “network spectatorship.” At this time, communication among netizens begins to show the characteristics of emotional transmission (64). Netizens exhibit distinct emotional tendencies toward different types of network public opinion on university emergencies (Figure 9). These emotions form a specific rendering atmosphere, which, in turn, affects the psychological and new media fields. Therefore, the diffusion of the social field is the accumulation of network public opinion on university emergencies into the outbreak period.

(3) Outbreak period

After the brewing and diffusion periods, network public opinion on university emergencies enters the outbreak period. The psychological and social fields are jointly involved in public opinion through the new media field, and the influx of public opinion and emotion in a short period of time creates a huge “storm of public opinion” (65). It can be argued that the outbreak is the result of all three fields working together.

(4) Dissipation period

When the network public opinion on university emergencies enters the dissipation period, the new media field dominates. The management department employs stringent measures to regulate and steer network public opinion on university emergencies and constrain the participants’ communication behavior in the psychological field. This leads to a gradual return of rationality in the emotional landscape of the social field, resulting in a sustained decline in the impact index of public opinion. Figure 9 shows the rapid quelling of public emotion during the dissipation period due to the positive public response and joint governance efforts of various departments. Conversely, if management fails to provide appropriate guidance, the decline in public opinion influence index will turn upward. This will induce a wider range of public opinion risks, produce a secondary public opinion crisis, and prolong the duration of public opinion. As shown in Figure 8, the impact index of public opinion on public services, mental health, and teacher–student safety peaked during the dissipation period. This implies that these three types of public opinion have a secondary public opinion in the dissipation period, which lengthens the duration of public opinion. Therefore, the dissipation period is critical for the governance of network public opinion on university emergencies.

(5) Calming period

In the calming period, public opinion is rarely mentioned. The psychological, social, and new media fields gradually return to an independent state, and the entire public opinion field withdraws from public view.

### 4.3 Governance strategies of network public opinion on university emergencies

Through an analysis of the causes, this study combines the evolution cycle of network public opinion on university emergencies to formulate governance strategies at three levels: public opinion prevention and management, in-process public opinion detection and control, and post-public opinion monitoring and evaluation.

(1) Public opinion prevention and management

Based on the results of the data analysis, this study formulates prevention and governance strategies for network public opinion on university emergencies from the aspects of subject, type, and time. First, the subjects of network public opinion on university emergencies include students, teachers, and universities. The ability to respond to public opinion should be improved according to the characteristics of the different subjects. For example, students have active minds but lack social experience; therefore, it is necessary to strengthen the ideological construction of this group (66). Teachers are under pressure and easily exhausted; therefore, it is necessary to strengthen psychological interventions for this group (67). As administrators, universities should actively enhance their sense of responsibility to realize the safe dissemination of network public opinion on university emergencies (68). Second, the analysis and early warning should be strengthened for network public opinion on the high-incidence categories (mental health and teacher–student safety) during high-incidence periods (July and December). Moreover, university management strategies should be optimized to prevent the emergence of network public opinion on university emergencies.

(2) In-process public opinion detection and control

During the brewing period, network public opinion on university emergencies is dominated by the psychological field, which is the best period for public opinion governance. At this time, public opinion governance should be centered on the university. The subject of governance should grasp the public opinion situation and guide the positive development of public opinion from two aspects. First, identifying the source of alarm and quickly judging the type of network public opinion on university emergencies. Second, analyzing the warning signs. The university community’s degree of attention to network public opinion on university emergencies can be determined using the degree of public opinion topics, degree of dissemination, and interaction rate as main indicators.

During the diffusion period, the network public opinion on university emergencies is dominated by the social field, which is the last period to prevent the outbreak of public opinion. At this time, the governance of public opinion should be conducted mainly by universities and supplemented by social opinion leaders. The governance subject should take hold of the power of public opinion discourse and control its spread in two ways. First, universities should take the initiative to establish a collaborative information dissemination system that encompasses their official website and opinion leaders. This system aims to respond to concerns regarding different types of network public opinion on university emergencies. Second, the governance subject should actively appease public emotions and prevent the large-scale contagion of negative emotions (69).

During the outbreak period, network public opinion on university emergencies is jointly influenced by psychological, social, and new media fields. Hence, multiple subjects should collaborate to conduct public opinion governance, which should control the risk of public opinion and reduce its negative impacts. First, a mechanism for leading public opinion ought to be established. Government-centralized leadership and unified deployment of risk governance for network public opinion on university emergencies are recommended. Furthermore, collaborative governance alliances should be broadened, coordination among key players strengthened, and the responsibilities of all parties involved refined. Second, an online–offline linkage mechanism ought to be built. Online and offline synergistic governance can be realized through the good interaction of multiple governance subjects.

During the dissipation period, network public opinion on university emergencies is dominated by the new media field with a declining impact index. This is a critical period for public opinion governance, when mainstream official media should be considered the center of public opinion governance. The governance subject should consolidate the results of the battle and prevent the occurrence of secondary public opinion crises. First, the implementation of public opinion governance should be emphasized. It is imperative to conduct real-time supervision to ensure the effective implementation of governance results pertaining to network public opinion on university emergencies and, second, emphasize positive opinion guidance, expand applications of communication tools, enhance public engagement, and steer network discourse on university emergencies positively.

(3) Post-public opinion monitoring and evaluation

During the calming period, the impact index of network public opinion on university emergencies tends to approach zero. At this time, the aftermath of public opinion should have universities at the center (70). First, post-evaluation work should be strengthened. Universities should organize the post-evaluation work of public opinion in a timely manner to learn from the experience. This is conducive to checking and mending the emergency response mechanism for network public opinion on university emergencies. Second, the positive publicity of universities should be strengthened. Universities should prioritize the establishment of ideological fortifications, cultivating a favorable image of themselves through the meticulous development of campus culture.

## 5 Method comparison

### 5.1 Comparative analysis of research methods

This study used three core methods to analyze the causes and governance strategies of network public opinion on university emergencies: data analysis, quantitative analysis, and theoretical inference. To gain a deeper understanding of the application value of these methods, this study compares them with the methods used in recent relevant studies.

(1) Data analysis versus empirical analysis. In the realm of social sciences, the majority of extant research predominantly employs empirical analysis to verify or reveal the correlations among social phenomena. For example, Ren utilizes the COVID-19 epidemic as an empirical case to validate hypotheses by observing public opinion evolution and analyzing interactions among communication elements (27). However, empirical analysis methods face challenges in excluding all confounding variables and factors, hindering causal relationship confirmation. Hence, this study adopts statistical, comparative, clustering, intersection, correlation, and other data analytical methods to systematically analyze the relevant public opinion data collected and processed from the Zhiwei Data Sharing Platform. Based on these data-driven methods, this study was able to further subdivide the types of network public opinion on university emergencies and discover the patterns in terms of time, subject, and emotion. Compared with the prior studies, this study can better grasp the interaction relationship between variables of public opinion elements.
(2) Quantitative analysis versus qualitative analysis. Most existing studies of network public opinion mainly use qualitative analysis to identify key factors and construct conceptual models. For example, Yang constructs a framework of factors for the emergence of network public opinion by qualitatively categorizing the comments (18). However, the results of qualitative analysis are inevitably limited by a series of assumptions and subjective emotions in practice, and different researchers may have different interpretations of the same phenomenon. Hence, this study uses visual quantitative analysis methods such as mathematics and statistics to visualize the data results through graphs and other means. These quantitative analysis methods further verified the existence of a distinct correlation among various elements of network public opinion, thereby providing a data basis for the subsequent construction of the network public opinion field of university emergencies. Compared with qualitative research, this study emphasizes numerical data and the results of the analysis are more objective and generalizable.
(3) Integration of multiple methods versus a single research method. In existing research on network public opinion on university emergencies, the application of a single research method is prevalent. For example, Ye only studied the influencing factors of college students’ willingness to spread network public opinion through quantitative analysis methods such as questionnaires (3). However, a single research method is insufficient to meet the increasingly convergent and interdisciplinary demands, limiting researchers’ ability to fully understand and address complex issues. Hence, this study adopts a research paradigm that combines data-driven with theoretically oriented. Data from the Zhiwei Data Sharing Platform are quantitatively analyzed to extract public opinion trends, which are then interpreted using the theory of network public opinion field to deduce causes. Compared to a single research method, the integration of multiple methods enhances the scientific, innovative, and comprehensive nature of research results.

### 5.2 Advantages of the proposed methods

Through the comparative analysis above, the characteristics and advantages of the method utilized by this study can be summarized to highlight the academic contributions of this study.

First, the application of the Zhiwei Data Sharing Platform makes it possible to analyze the data in a more in-depth way to explore the potential value of the data. The Zhiwei Data Sharing Platform uses big data analysis technology, offering real-time, high-accuracy data. This study collects network public opinion data on university emergencies from the Zhiwei Data Sharing Platform, which helps to better explore the patterns and correlations among the types of public opinion, subjects, and time and space. This lays the foundation for subsequent analysis of public opinion evolution laws and trends.

Second, accurately quantify the relationship between public opinion data and make the research results easy to understand through intuitive graphics. Throughout the various stages of data collection, processing, and analysis, quantitative analysis methods are employed to ensure the accuracy and objectivity of our findings. Additionally, this study visualizes public opinion analysis through charts and images, simplifying complex data for intuitive understanding and enabling deeper research.

Third, the combination of data-driven and theoretically oriented makes the research results more comprehensive and systematic. This study is data-driven by visualizing and analyzing the network public opinion data of 204 cases of university emergencies. It is also theoretically-oriented, using the theory of the network public opinion field to facilitate the construction of the network public opinion field of university emergencies. The combined method of data-driven and theory-oriented analysis integrates theory and practice, facilitating the explanation, prediction, and analysis of the causes of network public opinion on university emergencies.

## 6 Conclusion and implications

This study constructs a network public opinion field model of university emergencies based on visual analysis and the theory of the network public opinion field. Using the model as a foundation, it explores the causes of network public opinion on university emergencies and proposes corresponding governance.

### 6.1 Theoretical implications

First, this study further subdivided network public opinion on university emergencies. Few studies have classified network public opinion on university emergencies. Based on real cases and related studies, this study classified network public opinion on university emergencies into six types. It further confirmed that different types of network public opinion on university emergencies differ in terms of the occurrence, time of dissemination, impact index, and emotion of public opinion dissemination. The visualization results show that mental health and teacher–student safety are the most common types of network public opinion on university emergencies (83.3%). The emotional tendency expressed by the public is influenced by the type of public opinion and generally tends to be negative; 90.20% of the public opinions last for less than 19 days, and their influence ranges from 40 to 80.

Second, this study developed a network public opinion field model of university emergencies to elaborate the interactive relationship between the public opinion elements in the network field. From the perspective of the theory of the network public opinion field, the proposed network public opinion field model of university emergencies consists of subfields with different characteristics and operating rules. For the psychological field, public universities (88.24%) and students (48%) are important subjects of network public opinion on university emergencies, implicitly mirroring the underlying social structure and relationships shaping public opinion. In the social field, different kinds of university emergencies form a consensus of network public opinion within a certain social scope, leading to the spread of certain emotional tendencies (Figure 9). For the new media field, media technology, and communication channels affect the fluidity of public opinion information, with greater fluidity correlating to longer opinion durations.

Third, from the perspective of the entire lifecycle, this study comprehensively analyzed the causes and governance strategies of network public opinion on university emergencies. This study finds that the development of network public opinion on university emergencies conforms to the lifecycle theory, which contains five periods: brewing, diffusion, outbreak, dissipation, and calming. Most of the existing literature has focused on the impact of individual factors on the evolution of network public opinion on university emergencies. In contrast, this study provided a comprehensive analysis of the causes and trends in each period of public opinion development, enabling the development of tailored public opinion governance strategies for each period.

### 6.2 Practical implications

First, the university is the key subject in the governance of network public opinion on university emergencies. The network public opinion on university emergencies exhibits certain regularity. Therefore, universities should accurately grasp the key elements in the formation of university emergency network public opinion. In light of the high-incidence types and periods of network public opinion on university emergencies, it is imperative to establish an effective emergency response system. In addition, university administrators should uphold the principle of people-oriented public opinion guidance and emphasize students’ subjectivity. Therefore, ideological education activities should be conducted regularly to improve students’ fundamental network literacy.

Second, period-based governance helps improve the governance efficiency of network public opinion on university emergencies. The results of this study show that network public opinion on university emergencies presents different characteristics in each period. During the brewing period, the scope of public opinion is limited to within the university, and this is an important node for effectively containing its spread. During the diffusion period, potential secondary public opinion is a significant factor influencing the duration of public opinion. Preventing the occurrence of such phenomena is crucial for governance at this period. The calming period signifies a marked decrease or complete cessation of public opinion activities, serving as a symbolic node of the disappearance of public opinion. Formulating reasonable governance strategies based on period characteristics is conducive to prescribing the right remedy and controlling the spread of public opinion risks.

Third, building a pattern of collaborative governance by multiple subjects is necessary for governing network public opinion on university emergencies. Synthesizing this study reveals that the formation of network public opinion on university emergencies involves university groups, social netizens, social media, and other subjects. Different subjects play a corresponding role in promoting the evolution of public opinion. Therefore, it is necessary to not only highlight the joint participation of multiple subjects but also strengthen the subjects’ collaborative governance ability.

### 6.3 Limitations and future directions

In this study, only the Zhiwei Data Sharing Platform was used as the data source, which resulted in insufficient sample diversity. Further evidence is required to support the interaction between fields within the network public opinion field of university emergencies. In the future, different regions and levels of network public opinion on university emergencies will be selected to conduct extended research and realize the deep integration of theory and practice.

## Data Availability

The raw data supporting the conclusions of this article will be made available by the authors, without undue reservation.

## Footnotes

^1^ http://university.zhiweidata.com/
